# Ultrasonographic Evaluation of Asymptomatic Shoulder Involvement in Rheumatoid Arthritis: A Prospective Case–Control Study

**DOI:** 10.3390/jcm15103666

**Published:** 2026-05-10

**Authors:** Yunus Emre Doğan, Çiğdem Çınar, Kadriye Öneş, Burak Kütük, Muhsin Doran, Halil Harman

**Affiliations:** 1 Department of Physical Medicine and Rehabilitation, Fatih Sultan Mehmet Training and Research Hospital, University of Health Sciences, 34752 Istanbul, Türkiye; 2 Department of Physical Medicine and Rehabilitation, School of Medicine, University of Biruni, 34010 Istanbul, Türkiye; ccdem.inar@gmail.com; 3 Department of Physical Medicine and Rehabilitation, Physical Therapy and Rehabilitation Training and Research Hospital, University of Health Sciences, 34668 Istanbul, Türkiye; kadriyeones@yahoo.com (K.Ö.); burak01kutuk@gmail.com (B.K.); muhsin-doran@hotmail.com (M.D.); drhharman@yahoo.com (H.H.)

**Keywords:** rheumatoid arthritis, shoulder, ultrasonography, biceps tendinopathy, disease activity, subclinical involvement

## Abstract

**Background/Objectives:** Shoulder involvement is common in rheumatoid arthritis (RA), yet periarticular pathology may remain subclinical. Musculoskeletal ultrasonography enables detection of early inflammatory and structural changes; however, data on asymptomatic shoulder involvement in RA are limited. This study aimed to evaluate ultrasonographic shoulder findings in asymptomatic RA patients, compare them with healthy controls, and assess their association with disease activity. **Methods:** This prospective case–control study included 31 patients with RA without shoulder pain and 33 asymptomatic healthy controls. Bilateral shoulder examinations were performed using standardized ultrasonographic protocols. Periarticular findings were assessed using the Ultrasound Shoulder Pathology Rating Scale (USPRS), and acromiohumeral distance (AHD) was measured. Disease activity was evaluated using the Disease Activity Score in 28 joints (DAS28). Group comparisons, correlation analyses, receiver operating characteristic (ROC) analysis, and multivariate logistic regression were performed. **Results:** There were no significant differences in demographic characteristics or comorbidities between groups. Biceps tendinopathy scores were significantly higher in RA patients than in healthy controls (1.0 ± 0.79 vs. 0.5 ± 0.56, *p* = 0.01), whereas other ultrasonographic parameters, including supraspinatus tendinopathy, dynamic impingement findings, AHD, and total USPRS score, did not differ significantly. DAS28 showed significant positive correlations with biceps tendinosis (r = 0.37, *p* < 0.05) and supraspinatus tendinosis (r = 0.36, *p* < 0.05). Total USPRS demonstrated acceptable discrimination for identifying moderate–high disease activity (AUC = 0.73). In multivariate analysis, DAS28 was independently associated with biceps tendinopathy (OR = 1.62, 95% CI: 1.05–2.49). **Conclusions:** Asymptomatic patients with RA exhibit a higher burden of biceps tendon involvement compared with healthy individuals, and subclinical shoulder ultrasonographic findings are associated with disease activity. Asymptomatic patients with RA demonstrated a significantly higher biceps tendinopathy score compared with healthy controls, whereas most other ultrasonographic parameters, including total USPRS score and AHD, did not differ significantly. These findings suggest that the long head of the biceps tendon may represent a relatively more sensitive site of subclinical periarticular involvement in RA; however, the overall ultrasonographic differences were limited and should be interpreted cautiously.

## 1. Introduction

Rheumatoid arthritis (RA) is a chronic, systemic inflammatory disease characterized by persistent synovitis and progressive structural damage, leading to disability and reduced quality of life. Contemporary management strategies emphasize early diagnosis, close monitoring, and treatment adaptation to achieve remission or at least low disease activity, because uncontrolled inflammation is strongly linked to irreversible musculoskeletal damage and long-term functional impairment [1,2].

Although clinical attention in RA often focuses on small joints, large-joint and periarticular involvement can substantially contribute to disability. The shoulder is particularly relevant because it integrates complex rotator cuff and bursal biomechanics and is frequently exposed to inflammatory and degenerative processes. Importantly, shoulder involvement may remain clinically silent for prolonged periods, while subclinical inflammatory or tendon-related changes continue to evolve. In this setting, relying solely on symptoms and physical examination may delay recognition of early periarticular pathology and miss opportunities for timely therapeutic adjustment aligned with treat-to-target principles [3,4,5].

Musculoskeletal ultrasonography (US) has become a key imaging tool in rheumatology because it is accessible, repeatable, dynamic, and capable of visualizing both inflammatory and structural abnormalities. Practical frameworks and recommendations increasingly support the use of US to complement clinical assessment in RA—particularly for detecting subclinical inflammation, refining evaluation of disease activity, and assisting decision-making in routine practice [6,7,8,9,10,11]. Moreover, EULAR guidance has emphasized transparent and standardized reporting of US studies in rheumatic and musculoskeletal diseases to improve reproducibility and interpretability across centers [7,12,13,14].

Standardization of scanning technique is also essential for shoulder US, given the joint’s complex anatomy and the operator dependence of ultrasound. The European Society of Musculoskeletal Radiology (ESSR) provides structured technical guidance for shoulder scanning positions and stepwise evaluation of key periarticular structures, including the long head of the biceps tendon and rotator cuff tendons [15]. Such standardized protocols are particularly valuable in research designs comparing subtle subclinical findings between patient groups.

A critical methodological consideration in studies of “asymptomatic” shoulder involvement is that ultrasonographic abnormalities may also be observed in the general population, even in the absence of pain. For example, population-based investigations have demonstrated a non-trivial prevalence of asymptomatic shoulder US findings among working-age adults, underscoring the necessity of an appropriately matched healthy control group and structured scoring approaches when attributing abnormalities to RA-related mechanisms [16,17,18,19,20].

Within RA populations, emerging evidence suggests that shoulder US findings may relate to systemic disease activity rather than pain alone [21,22,23,24,25]. A pilot study reported associations between shoulder US abnormalities and disease activity measures in RA, supporting the concept that periarticular shoulder changes can reflect the broader inflammatory burden even when clinical symptoms are limited or absent [26]. However, the literature on asymptomatic shoulder involvement in RA remains relatively sparse and heterogeneous with respect to scanning protocols, outcome definitions, and scoring methods, leaving uncertainty about which subclinical abnormalities are most discriminative and how closely they track disease activity [27,28,29,30].

To address these gaps, the present prospective single-blind case–control study aimed to evaluate ultrasonographic shoulder involvement in rheumatoid arthritis (RA) patients without shoulder pain and to compare these findings with those of asymptomatic healthy individuals using a standardized scanning protocol. By incorporating structured assessments of tendon pathology and dynamic impingement evaluation, together with acromiohumeral distance (AHD) measurement as a sonographic marker of the subacromial space and rotator cuff-related mechanics, this study sought to characterize the spectrum of subclinical periarticular shoulder abnormalities in RA and to explore their relationship with disease activity. We hypothesized that patients with RA would exhibit a greater burden of subclinical tendon-related pathology—particularly involving the long head of the biceps tendon—and that these ultrasonographic findings would correlate with established disease activity indices. Through this approach, the study aimed to highlight the diagnostic value of shoulder ultrasonography in the early detection of inflammatory changes and its potential role in optimizing disease monitoring and management in RA.

## 2. Materials and Methods

### 2.1. Study Design

This prospective, single-blind, case–control study was approved by the Institutional Review Board of Bakırköy Dr. Sadi Konuk Training and Research Hospital (Decision No.: 2023-20-10). The study protocol was registered at ClinicalTrials.gov (NCT06572696). All procedures were conducted in accordance with the Declaration of Helsinki, and written informed consent was obtained from all participants.

**Figure 1:**
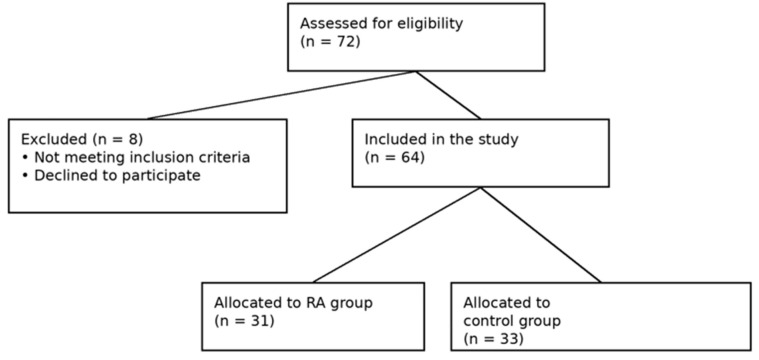
Flowchart of the study.

A single-blind design was used: the physician performing the standardized ultrasound (US) examinations was blinded to group allocation and clinical data, while the clinical shoulder examination was conducted by a different physician. The flow of participant recruitment, exclusion, and group allocation is summarized in Figure 1.

### 2.2. Participants and Clinical Assessment

Patients were consecutively recruited according to the order of outpatient clinic attendance, irrespective of age, sex, or disease activity. Patients aged 18–75 years with a diagnosis of RA according to the 2010 ACR/EULAR classification criteria were eligible [13,31]. Additional inclusion criteria were disease duration ≥ 12 months and absence of shoulder pain or shoulder-related complaints at enrollment. Exclusion criteria were suspected clinical shoulder pathology, previous shoulder surgery, direct shoulder trauma, history of infectious arthritis, other connective tissue diseases, serious chronic somatic or psychiatric disease, any fracture affecting the upper extremity, or limitation of shoulder range of motion.

Healthy volunteers without shoulder pain/complaints were recruited and matched to the RA group for age and sex distribution, and for non-RA comorbidities (e.g., hypertension, diabetes). The same exclusion criteria were applied.

Demographic data (age, sex, height, weight, BMI, dominant hand, marital and educational status, occupation) and comorbidities were recorded. For RA participants, disease duration and medication history (DMARDs, biologics, corticosteroids) were documented. Serological data, including rheumatoid factor (RF) and anti-cyclic citrullinated peptide (anti-CCP) antibody status, were not systematically available for all patients; therefore, serological subgroup analyses were not performed. HLA-B27 testing was not performed routinely, as all patients were diagnosed with rheumatoid arthritis according to the 2010 ACR/EULAR classification criteria and did not have clinical features suggestive of spondyloarthritis.

Disease activity was evaluated using the Disease Activity Score in 28 joints (DAS28), which integrates tender and swollen joint counts, patient global assessment (VAS), and an acute-phase reactant [32]. A standard clinical shoulder examination was performed in all participants, including assessment of active/passive range of motion and commonly used special tests for tendon pathology/impingement. Range of motion was assessed clinically; however, no standardized goniometric measurements were performed. To assess intra-rater reliability, repeated US measurements were obtained from a subset of participants on two occasions separated by 24 h. Reliability was quantified using intraclass correlation coefficients (ICCs). Detailed joint-specific involvement beyond DAS28 components was not analyzed for correlation with shoulder ultrasonographic findings. Hand grip strength was not specifically measured or analyzed in relation to shoulder ultrasonographic findings.

### 2.3. Ultrasonographic Evaluation and Outcomes

Bilateral shoulder US examinations were performed by a single experienced operator (>5 years of musculoskeletal US experience) using an Esaote MyLab60 system (Esaote SpA, Genoa, Italy) with a 7–12 MHz linear transducer. The operator was blinded to clinical findings and group allocation. A standardized shoulder scanning protocol was used in accordance with the European Society of Musculoskeletal Radiology (ESSR) technical guidelines. The long head of the biceps tendon was examined with the participant seated, elbow flexed at 90°, and forearm supinated. The subscapularis tendon was assessed with slight external rotation. The supraspinatus tendon was evaluated in longitudinal and transverse planes with the shoulder positioned in internal rotation (hand toward the back pocket), consistent with standardized shoulder US positioning [15,33]. Although ultrasonographic findings such as effusion and synovitis were assessed qualitatively within the USPRS framework, subgroup analyses based on their presence or absence were not performed.

### 2.4. Ultrasound Shoulder Pathology Rating Scale (USPRS)

Periarticular structural and dynamic abnormalities were graded using the Ultrasound Shoulder Pathology Rating Scale (USPRS) [34]. This scale was selected because it enables standardized assessment of both static tendon pathology and dynamic impingement phenomena, which are relevant to periarticular involvement in inflammatory rheumatic diseases. USPRS comprises static assessments (biceps tendinopathy, supraspinatus tendinopathy, and tuberculum majus cortical irregularity) and dynamic assessments (supraspinatus impingement during abduction and subscapularis impingement during external rotation). The total USPRS score (range 0–20) was calculated by summing component scores, with higher scores indicating greater pathology burden [35]. For between-group comparisons, measurements obtained from the dominant shoulder were used to ensure analytical consistency and to avoid within-subject dependency.

### 2.5. Acromiohumeral Distance (AHD)

AHD was measured as a sonographic marker of the subacromial space. Measurement technique and interpretive rationale followed established approaches in the literature on AHD reliability and clinical applicability [36,37]. Participants were examined in a neutral resting position with a standardized arm/elbow posture. The transducer was placed longitudinally at the lateral edge of the acromion, and the distance between the inferior acromial cortex and the humeral head was recorded in millimeters.

**Figure 2:**
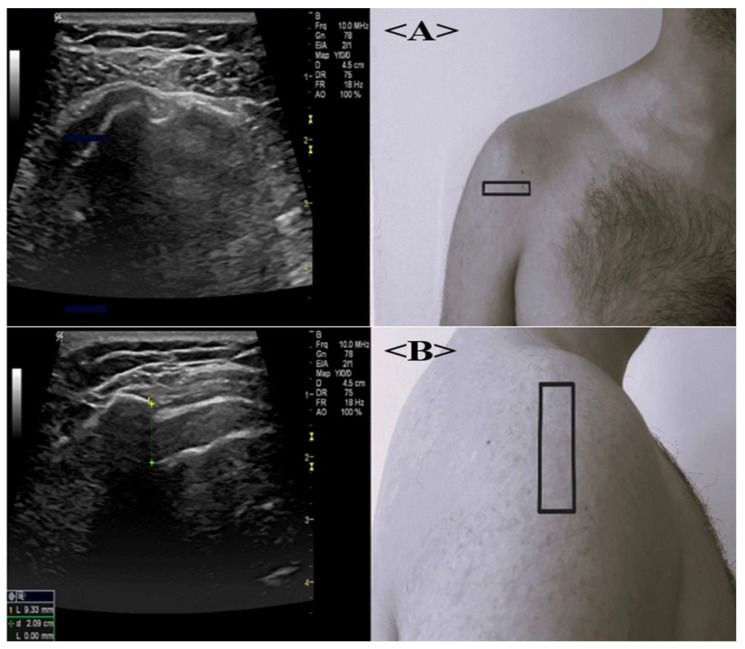
(**A**) Ultrasonographic image of the biceps tendon of a 56-year-old male patient with RA. (**B**) Ultrasonographic image of the acromiohumeral distance (AHD) measurement. The black rectangular boxes on the clinical photographs indicate the approximate transducer placement and anatomical region evaluated during ultrasonographic examination. The colored line and caliper markers on the ultrasound image in panel (**B**) represent the AHD measurement.

The long head of the biceps tendon was evaluated in transverse and longitudinal planes with the patient seated and the elbow flexed at 90°, and the acromiohumeral distance (AHD) was measured by placing the transducer longitudinally at the lateral border of the acromion, as illustrated in Figure 2A,B.

### 2.6. Statistical Analysis

Statistical analyses were performed using SPSS version 27.0 (IBM Corp., Armonk, NY, USA). A priori sample size estimation was performed using GPower (version 3.1) based on α = 0.05 and 95% power. Descriptive statistics were reported as mean ± SD for continuous variables and *n* (%) for categorical variables. Normality was assessed using the Kolmogorov–Smirnov test. Between-group comparisons were performed using the Mann–Whitney U test for non-normally distributed continuous variables and the chi-square test (or Fisher’s exact test when appropriate) for categorical variables. Within-group paired comparisons (dominant vs. non-dominant shoulder) were evaluated using the Wilcoxon signed-rank test. Correlations between ultrasonographic findings and DAS28 were assessed using Spearman’s correlation coefficient. Given the exploratory nature of the study and the relatively small sample size, no formal correction for multiple comparisons was applied; therefore, the results should be interpreted with caution. A two-sided *p* value of <0.05 was considered statistically significant.

## 3. Results

Demographic and clinical characteristics of RA and healthy control groups were shown in Table 1. A total of 31 patients with RA and 33 healthy controls were included. There were no statistically significant differences between the groups in terms of age, gender distribution, hand dominance, or comorbid conditions, including hypertension, diabetes mellitus, cardiac disease, and thyroid disease (all *p* > 0.05). The mean disease duration in the RA group was 116.3 ± 93.3 months. Among RA patients, 83.9% were receiving conventional disease-modifying antirheumatic drugs (DMARDs), 41.9% biological agents, and 38.7% corticosteroids. The mean DAS28 score was 3.6 ± 1.46, and the mean VAS score was 45.8 ± 29.9 (Table 1).

Comparison of USPRS and AHD in dominant and non-dominant shoulders of patients with RA is shown in Table 2. No statistically significant differences were observed between dominant and non-dominant shoulders with respect to biceps tendinopathy, supraspinatus tendinopathy, tuberculum majus cortical irregularity, dynamic supraspinatus impingement, dynamic subscapularis impingement, total USPRS score, or acromiohumeral distance (all *p* > 0.05) (Table 2).

Comparison of dominant-side USPRS and AHD in healthy control and RA patients is shown in Table 3. Biceps tendinopathy scores were significantly higher in RA patients compared with healthy controls (1.0 ± 0.79 vs. 0.5 ± 0.56, *p* = 0.01). No statistically significant differences were observed between the groups with respect to supraspinatus tendinopathy, tuberculum majus cortical irregularity, dynamic supraspinatus or subscapularis impingement, total USPRS score, or acromiohumeral distance (all *p* > 0.05) (Table 3).

**Figure 3:**
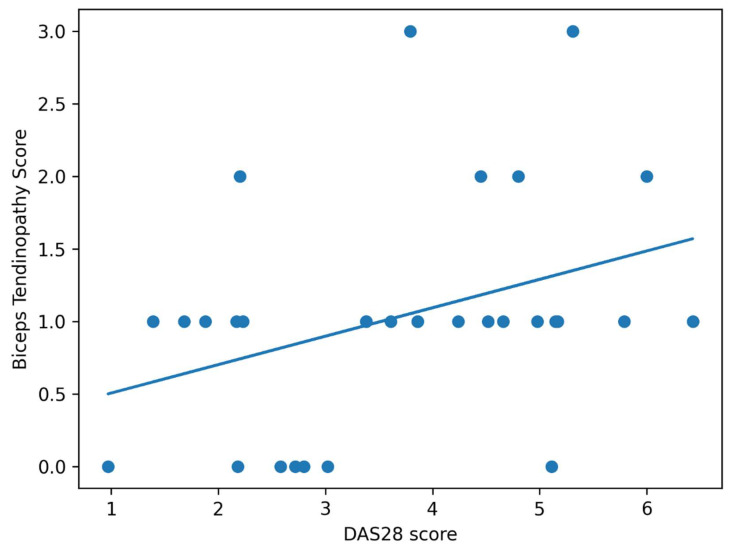
Correlation between DAS28 and biceps tendinopathy. Scatter plot showing the relationship between DAS28 score and biceps tendinopathy severity. Blue dots represent individual patient data points, and the blue solid line represents the linear regression trend line demonstrating a significant positive correlation (r = 0.37, *p* = 0.04).

Correlations between shoulder joint pathologies and DAS28 Score in RA patients were shown in Table 4. The DAS28 score showed a moderate positive correlation with biceps tendinosis (r = 0.37, *p* < 0.05) and supraspinatus tendinosis (r = 0.36, *p* < 0.05). In addition, biceps tendinosis was significantly correlated with supraspinatus tendinosis (r = 0.52, *p* < 0.01) and tuberculum majus cortical irregularity (r = 0.44, *p* < 0.05). Supraspinatus tendinosis demonstrated strong correlations with tuberculum majus cortical surface irregularity (r = 0.79, *p* < 0.01), dynamic supraspinatus impingement (r = 0.58, *p* < 0.01), and dynamic subscapularis impingement (r = 0.59, *p* < 0.01). No statistically significant correlation was observed between DAS28 score and tuberculum majus cortical irregularity or dynamic impingement parameters (*p* > 0.05) (Table 4, Figure 3).

**Figure 4:**
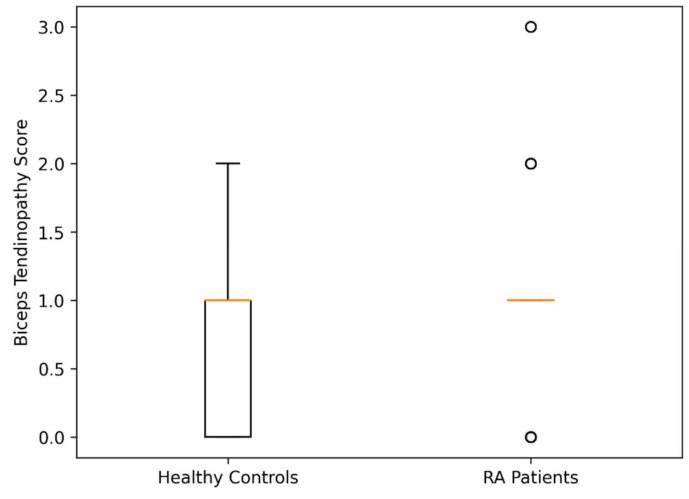
Boxplot of biceps tendinopathy scores. Boxplot comparison of biceps tendinopathy scores between RA patients and healthy controls. RA patients demonstrated significantly higher scores (*p* = 0.01). The boxes represent the interquartile range (IQR), the horizontal line within each box represents the median value, whiskers indicate the range of non-outlier values, and open circles represent outlier observations.

Effect size analysis of ultrasonographic findings between groups is shown in Table 5. Biceps tendinopathy demonstrated a moderate effect size (Cohen’s d = 0.70). Tuberculum majus cortical irregularity and total USPRS score showed small-to-moderate effect sizes (Cohen’s d = 0.48 and 0.45, respectively), whereas supraspinatus tendinopathy and dynamic subscapularis impingement exhibited small effect sizes (Cohen’s d = 0.35 and 0.36). Dynamic supraspinatus impingement demonstrated a negligible effect size (Cohen’s d = −0.12). While biceps tendinopathy demonstrated a moderate effect size (Cohen’s d = 0.70), the absolute difference between groups remained relatively small. Other ultrasonographic parameters showed small or negligible effect sizes, suggesting limited between-group separation for these variables (Table 5, Figure 4).

**Figure 5:**
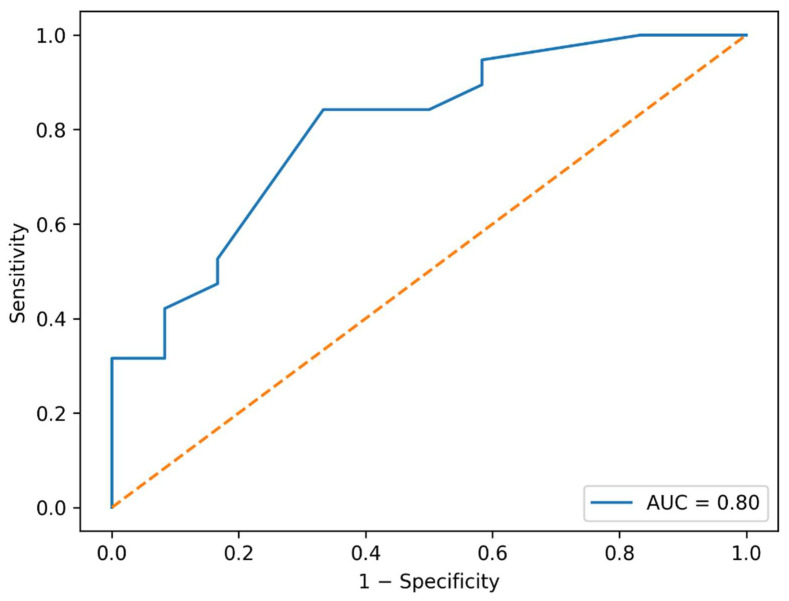
ROC Curve for Total USPRS in Predicting Moderate–High Disease Activity. ROC curve demonstrating the diagnostic performance of total USPRS score for identifying moderate–high disease activity (DAS28 ≥ 3.2).

ROC analysis of ultrasonographic parameters for identifying moderate–high disease activity was shown in Table 6. The total Ultrasound Shoulder Pathology Rating Scale (USPRS) score demonstrated acceptable discriminative ability, with an area under the curve (AUC) of 0.73 (95% CI: 0.58–0.89), sensitivity of 71%, and specificity of 75% at an optimal cut-off value of ≥4. Biceps tendinopathy score also showed moderate diagnostic performance (AUC = 0.71, 95% CI: 0.55–0.87), whereas supraspinatus tendinopathy demonstrated slightly lower accuracy (AUC = 0.68, 95% CI: 0.51–0.84). In contrast, acromiohumeral distance (AHD) showed poor discriminative ability for disease activity (AUC = 0.54, 95% CI: 0.38–0.70) (Table 6, Figure 5).

Multivariate logistic regression analysis for biceps tendinopathy was shown in Table 7. Disease activity, assessed by DAS28, was independently associated with the presence of biceps tendinopathy, with each 1-unit increase in DAS28 corresponding to a 62% increase in odds (OR = 1.62, 95% CI: 1.05–2.49, *p* = 0.03). In contrast, disease duration, age, and steroid use were not significantly associated with biceps tendinopathy in the multivariate model (*p* > 0.05 for all) (Table 7).

Intra-rater reliability of ultrasonographic evaluation performed by the same physician at different times. All evaluations were found to be highly reliable (0.80 ≤ Cronbach’s Alpha). In addition, the absolute and relative values of measurement technical error according to AHD were calculated as 0.06 mm and 0.59%, respectively, and the reliability of measurements was at an acceptable level (less than 5%).

## 4. Discussion

In this prospective single-blind case–control study, the principal between-group difference was confined to biceps tendinopathy, which was significantly more pronounced in asymptomatic RA patients than in healthy controls. In contrast, supraspinatus tendinopathy, tuberculum majus cortical irregularity, dynamic impingement parameters, AHD, and total USPRS score did not differ significantly between groups. Therefore, the findings should not be interpreted as evidence of generalized ultrasonographic shoulder involvement in asymptomatic RA patients, but rather as indicating a selective signal predominantly involving the long head of the biceps tendon.

Meroni et al. demonstrated that ultrasound abnormalities can be common even among asymptomatic individuals, emphasizing that imaging findings must be interpreted against an appropriate control group rather than assumed pathologic by default [16]. Tran et al. similarly highlighted that imaging-detected shoulder pathologies do not consistently map onto symptoms or symptom persistence, reinforcing that “abnormal” imaging may reflect background structural variation, age-related changes, or subclinical tissue adaptation rather than pain-generating pathology [28]. Our case–control design directly addresses this challenge by comparing asymptomatic RA patients with asymptomatic controls under a standardized scanning framework, and the selective between-group signal we observed (biceps tendinopathy) suggests that certain tendon-related findings may be more disease-relevant than others in this clinical context.

Elbinoune et al. reported a high prevalence of ultrasound abnormalities in the rheumatoid shoulder and emphasized the ability of musculoskeletal ultrasound to detect subclinical involvement [24]. Leblebicier et al. found that shoulder ultrasound findings in RA could be associated with disease activity, supporting a link between systemic inflammation and periarticular shoulder pathology [26]. Our results extend these observations by focusing on patients without shoulder pain and by demonstrating that biceps tendinopathy differs from controls and correlates with DAS28, including in multivariable analysis. Anatomically, the long head of the biceps tendon is closely related to the glenohumeral joint and is surrounded by a synovial sheath; therefore, it may plausibly reflect synovitis-driven peri-tendinous inflammatory processes earlier than some other rotator cuff structures. This provides a biologically coherent explanation for why biceps tendinopathy emerged as the most discriminative ultrasonographic parameter in our asymptomatic cohort. Although shoulder involvement with tenosynovitis and enthesitis may resemble features seen in spondyloarthritis, all patients in this study fulfilled established RA classification criteria, reducing the likelihood of diagnostic overlap.

Leblebicier et al. reported associations between shoulder US findings and disease activity measures, suggesting that higher inflammatory burden may be mirrored by increased shoulder pathology [26]. Our study is consistent with this framework, showing statistically significant but modest correlations between DAS28 and both biceps and supraspinatus tendinosis, and additionally showing that DAS28 independently predicts biceps tendinopathy (OR 1.62 per one-unit increase). Clinically, these results align with treat-to-target principles, where ongoing inflammatory activity is expected to manifest across musculoskeletal structures even before symptoms develop [1]. This may support using focused shoulder ultrasonography—particularly assessment of the biceps tendon—as a complementary tool in patients with moderate–high disease activity, even when shoulder pain is absent. However, it is important to emphasize that the observed correlations (r ≈ 0.36–0.37) are relatively weak and indicate only modest associations between disease activity and tendon pathology. These findings should not be interpreted as evidence of a strong or direct relationship. Instead, they suggest that subclinical shoulder involvement may partially reflect systemic inflammatory burden, but is likely influenced by multiple additional factors. Therefore, the clinical significance of these correlations should be interpreted cautiously, and causal inferences cannot be drawn from this cross-sectional analysis. Future studies should investigate whether involvement of specific joints may predict subclinical shoulder abnormalities.

Meroni et al. demonstrated substantial prevalence of asymptomatic findings in the general population, implying that certain ultrasound abnormalities (e.g., mild degenerative changes) may have limited specificity for inflammatory disease when studied cross-sectionally [16]. Tran et al. similarly reported heterogeneous and often weak associations between imaging abnormalities and symptoms, suggesting that not all imaging findings represent clinically meaningful disease processes [28]. In our study, the lack of significant between-group differences for supraspinatus tendinopathy, tuberculum majus cortical irregularity, and dynamic impingement may therefore reflect a combination of background prevalence of such findings, limited incremental structural change in an asymptomatic cohort, and/or insufficient separation between groups for these endpoints. Importantly, effect size analysis suggested small-to-moderate differences for some non-significant parameters (e.g., total USPRS and tuberculum majus irregularity), which may indicate that clinically relevant differences could emerge more clearly in larger samples or longitudinal designs. Although biceps tendinopathy reached statistical significance with a moderate effect size (Cohen’s d = 0.70), the absolute difference between groups was relatively small, which may limit its immediate clinical interpretability. Moreover, most other ultrasonographic parameters demonstrated small or negligible effect sizes, indicating limited practical separation between asymptomatic RA patients and healthy controls. These findings suggest that, despite statistical significance, the overall clinical impact of the observed differences is modest. Therefore, the results should be interpreted cautiously, and the role of shoulder ultrasonography in this context may be better considered as complementary and exploratory rather than directly practice-changing.

McCreesh et al. evaluated reliability considerations for AHD measurement and highlighted that measurement methodology influences interpretability [36]. In our cohort, AHD did not differ between groups and performed poorly for discriminating moderate–high disease activity. This suggests that AHD—often discussed in the context of rotator cuff disease and subacromial impingement—may be less sensitive to early inflammatory periarticular changes in RA when pain is absent, and may be more reflective of mechanical or degenerative factors than inflammatory disease activity.

D’Agostino et al. proposed pragmatic algorithms for integrating ultrasound into RA management and emphasized that ultrasound can add value for disease assessment and monitoring when used judiciously [6]. Our ROC analysis showed that total USPRS (AUC 0.73) and biceps tendinopathy (AUC 0.71) had acceptable but moderate discrimination for identifying moderate–high disease activity, whereas AHD did not. While these AUC values do not imply standalone diagnostic capability, they support the idea that a concise shoulder ultrasound assessment—particularly incorporating biceps evaluation—may serve as an adjunct signal of inflammatory burden in selected patients. In practice, this could be most useful when clinical examination is unremarkable but systemic disease activity remains moderate–high. Although total USPRS and biceps tendinopathy demonstrated acceptable discriminative ability (AUC ≈ 0.7), this level of performance is generally considered moderate and insufficient for use as a standalone diagnostic or monitoring tool in clinical practice. These findings indicate that shoulder ultrasonography, in this context, should not be relied upon independently for disease activity assessment. Instead, it may provide complementary information when interpreted alongside established clinical indices such as DAS28. Therefore, the diagnostic utility of these ultrasonographic parameters should be viewed as supportive rather than definitive.

Costantino et al. provided EULAR recommendations for reporting ultrasound studies in rheumatic diseases, emphasizing standardized acquisition, definitions, and transparent reporting to support reproducibility [7]. Sconfienza et al. updated ESSR consensus indications for musculoskeletal ultrasound and reinforced the clinical relevance of ultrasound across multiple joints, including the shoulder [15]. Our use of a standardized scanning protocol, blinding, and intra-rater reliability assessment strengthens internal validity and aligns with these expectations, which should support interpretability of the findings across settings.

Smolen et al. emphasized that modern RA management depends on early identification of inflammatory activity and structured monitoring to prevent structural progression and disability [1]. Bilberg et al. demonstrated that shoulder function can be impaired early in RA, supporting the importance of recognizing shoulder involvement as part of comprehensive disease burden rather than as an isolated pain complaint [29]. In this context, our findings suggest that shoulder ultrasonography may be considered even in asymptomatic patients—particularly those with higher DAS28—to detect subclinical tendon involvement that may otherwise be missed.

From a clinical perspective, the findings of this study should be interpreted with caution. Although subclinical biceps tendon abnormalities were more frequent in asymptomatic RA patients and were associated with disease activity, the present study does not demonstrate whether these findings predict future shoulder symptoms, influence treatment decisions, or affect long-term outcomes. Therefore, routine screening of asymptomatic shoulders using ultrasonography cannot be recommended based on the current data alone. However, these findings suggest that targeted shoulder ultrasonography may have potential value in selected patients with moderate-to-high disease activity, particularly when clinical assessment is inconclusive. In this context, ultrasonographic evaluation of the long head of the biceps tendon may serve as an adjunctive tool for identifying subclinical periarticular involvement. Future longitudinal studies are required to determine the prognostic significance and clinical utility of these findings.

This study has several limitations. First, the single-center design and relatively small sample size (*n* = 64) may limit the generalizability of the findings and reduce statistical power, particularly for detecting subtle differences across multiple ultrasonographic parameters. Moreover, multiple statistical comparisons were performed without formal correction for multiplicity, which may increase the risk of type I error; therefore, some statistically significant findings should be interpreted with caution. Second, the control group did not include inflammatory laboratory markers, limiting direct comparisons of systemic inflammation between groups. In addition, serological subgroup analyses based on RF and anti-CCP status were not available for all patients, and HLA-B27 status was not evaluated. Although all patients fulfilled established RA classification criteria and did not exhibit clinical features of spondyloarthritis, these factors may limit interpretation of phenotype-specific ultrasonographic patterns. Third, the cross-sectional design precludes inference about prognostic significance. Furthermore, correlations between shoulder ultrasonographic findings and involvement of other specific joints were not analyzed, limiting understanding of systemic joint involvement patterns. Range of motion was not quantified using standardized goniometric measurements, and correlations between ultrasonographic findings and subtle functional limitations could not be assessed. Similarly, functional parameters such as hand grip strength were not evaluated. Finally, all ultrasonographic assessments were performed by a single experienced operator. Although intra-rater reliability was high, the absence of interobserver validation may limit the generalizability of the findings to broader clinical settings. In addition, subgroup analyses comparing RA patients with and without specific ultrasonographic features such as effusion or synovitis were not performed, which may limit more detailed interpretation of intra-group variability. Taken together, these considerations suggest that the results should be viewed as exploratory and hypothesis-generating rather than definitive. Future studies with larger, multicenter cohorts, predefined statistical correction strategies, and longitudinal follow-up are warranted to confirm these findings and to determine whether subclinical biceps abnormalities predict subsequent shoulder symptoms, structural progression, or functional outcomes.

## 5. Conclusions

In conclusion, asymptomatic RA patients demonstrated higher biceps tendinopathy scores compared with asymptomatic healthy controls, whereas most other ultrasonographic parameters, including total USPRS score and AHD, were not significantly different. These findings suggest that the long head of the biceps tendon may be a relatively sensitive site for detecting selective subclinical periarticular involvement in RA. Although a moderate effect size was observed for biceps tendinopathy, the relatively small absolute differences and generally low effect sizes across other parameters suggest limited immediate clinical impact. The limited between-group differences and moderate AUC values indicate that shoulder ultrasonography should be considered a complementary, hypothesis-generating tool rather than a standalone diagnostic or monitoring method in asymptomatic RA patients.

## Figures and Tables

**Table 1:** Demographic and clinical characteristics of RA and healthy control groups.

Variables	RA Patients	Healthy Control	*p* Value
	Mean ± SD or *n* (%)		
Age	56.0 ± 13.7	53.8 ± 7.5	0.090
Female gender	26 (83.9)	27 (81.8)	1.000
Male gender	5 (16.1)	6 (18.2)	
Right hand dominant	29 (93.5)	31 (93.9)	1.000
Left hand dominant	2 (6.5)	2 (6.1)	
Hypertension	10 (32.3)	8 (24.2)	0.581
Diabetes Mellitus	5 (16.1)	7 (21.2)	0.752
Cardiac Disease	4 (12.9)	4 (12.1)	1.000
Thyroid Disease	3 (9.7)	4 (12.1)	1.000
RA average disease duration (months)	116.3 ± 93.27	-	-
Using DMARD	*n* = 26 (83.9)	-	-
Using biological drugs	*n* = 13 (41.9)	-	-
Using DMARD + biological	*n* = 10 (32.3)	-	-
Using steroids	*n* = 12 (38.7)	-	-
Sedimentation	29.0 ± 29.18	-	-
CRP	12.2 ± 26.03	-	-
VAS	45.8 ± 29.86	-	-
DAS28	3.6 ± 1.46	-	-

SD: standard deviation; VAS: Visual Analog Scale; DAS28: Disease Activity Score in 28 joints; DMARD: disease-modifying antirheumatic drug.

**Table 2:** Comparison of USPRS and AHD in dominant and non-dominant shoulders of patients with RA.

Variables	Dominant Shoulder	Non-Dominant Shoulder	*p* Value
	Mean ± SD		
Biceps Tendinopathy	1.0 ± 0.79	1.0 ± 0.75	0.968
Supraspinatus Tendinopathy	1.3 ± 1.66	1.3 ± 1.25	0.520
Tuberculum Majus Cortical Irregularity	1.0 ± 1.04	1.1 ± 0.93	0.568
Dynamic Supraspinatus Impingement Assessment	0.5 ± 0.88	0.3 ± 0.65	0.473
Dynamic Subscapularis Impingement Assessment	0.4 ± 0.67	0.2 ± 0.57	0.430
Total Score	4.4 ± 4.13	4.1 ± 3.00	0.430
Acromiohumeral Distance (mm)	10.2 ± 1.58	10.5 ± 1.60	0.552

USPRS: Ultrasound Shoulder Pathology Rating Scale, AHD: acromiohumeral distance, mm: millimeter.

**Table 3:** Comparison of Dominant-Side USPRS and AHD in Healthy Control and RA Patients.

Variables	Healthy Control	RA Patients	*p* Value
	Mean ± SD		
Biceps Tendinopathy	0.5 ± 0.56	1.0 ± 0.79	0.010
Supraspinatus Tendinopathy	0.84 ± 0.66	1.3 ± 1.66	0.569
Tuberculum Majus Cortical Irregularity	0.6 ± 0.49	1.0 ± 1.04	0.179
Dynamic Supraspinatus Impingement	0.6 ± 0.65	0.5 ± 0.88	0.243
Dynamic Subscapularis Impingement	0.2 ± 0.43	0.4 ± 0.67	0.242
Total Score	3 ± 1.43	4.4 ± 4.13	0.317
Acromiohumeral distance (AHD)	10.4 ± 1.41	10.2 ± 1.58	0.767

**Table 4:** Correlations Between Shoulder Joint Pathologies and DAS28 Score in RA Patients.

Variables	DAS28	Biceps Tendinosis	Supraspinatus Tendinosis	Tuberculum Majus Cortical Surface	Dynamic Supraspinatus Impingement	Dynamic Subscapularis Impingement
DAS28	1.00					
Biceps tendinosis	0.371 *	1.00				
Supraspinatus tendinosis	0.362 *	0.523 **	1.00			
Tuberculum majus cortical surface	0.093	0.443 *	0.787 **	1.00		
Dynamic Supraspinatus impingement	0.104	0.306	0.578 **	0.638 **	1.00	
Dynamic Subscapularis impingement	0.320	0.569 **	0.589 **	0.409 *	0.284	1.00

Spearman correlation analysis. * *p* < 0.05, ** *p* < 0.01.

**Table 5:** Effect size analysis of ultrasonographic findings between groups).

Parameter	RA (Mean ± SD)	Control (Mean ± SD)	Mean Difference	Cohen’s d
Biceps tendinopathy	1.0 ± 0.79	0.5 ± 0.56	+0.5	0.70
Supraspinatus tendinopathy	1.3 ± 1.66	0.84 ± 0.66	+0.46	0.35
Tuberculum majus irregularity	1.0 ± 1.04	0.6 ± 0.49	+0.4	0.48
Dynamic supraspinatus impingement	0.5 ± 0.88	0.6 ± 0.65	−0.1	−0.12
Dynamic subscapularis impingement	0.4 ± 0.67	0.2 ± 0.43	+0.2	0.36
Total USPRS	4.4 ± 4.13	3.0 ± 1.43	+1.4	0.45

Effect sizes were calculated using Cohen’s d. Values of 0.2, 0.5, and 0.8 represent small, moderate, and large effects, respectively.

**Table 6:** ROC Analysis of Ultrasonographic Parameters for Identifying Moderate–High Disease Activity.

Parameter	AUC (95% CI)	Cut-Off	Sensitivity (%)	Specificity (%)	*p* Value
Biceps tendinopathy score	0.71 (0.55–0.87)	≥1	68	72	0.02
Supraspinatus tendinopathy	0.68 (0.51–0.84)	≥1	64	69	0.04
Total USPRS	0.73 (0.58–0.89)	≥4	71	75	0.01
AHD	0.54 (0.38–0.70)	≤10 mm	52	55	0.62

Cut-off values were determined using the Youden index.

**Table 7:** Multivariate logistic regression analysis for biceps tendinopathy.

Variable	OR	95% CI	*p* Value
DAS28 (per 1-unit increase)	1.62	1.05–2.49	0.030
Disease duration (years)	1.01	0.99–1.03	0.210
Age	1.02	0.97–1.07	0.410
Steroid use (yes/no)	1.28	0.62–2.64	0.490

Variables were selected based on clinical relevance. Model fit assessed using Hosmer–Lemeshow test.

## Data Availability

The datasets used and/or analyzed during the current study are available from the corresponding author upon reasonable request.

## Abbreviations

RA	Rheumatoid arthritis
US	Ultrasonography
USPRS	Ultrasound Shoulder Pathology Rating Scale
AHD	Acromiohumeral distance
DAS28	Disease Activity Score in 28 joints
VAS	Visual Analog Scale
DMARD	Disease-modifying antirheumatic drug
bDMARD	Biological disease-modifying antirheumatic drug
BMI	Body mass index
ICC	Intraclass correlation coefficient
ROC	Receiver operating characteristic
AUC	Area under the curve
OR	Odds ratio
CI	Confidence interval
ESSR	European Society of Musculoskeletal Radiology
